# Supplementary figures and images for: Relationships among barodontalgia prevalence, altitude, stress, dental care frequency, and barodontalgia awareness: a survey of Turkish pilots
Source: PeerJ. 2024 Apr 19;12:e17290. doi: 10.7717/peerj.17290 (PMC11034504; doi:10.7717/peerj.17290)

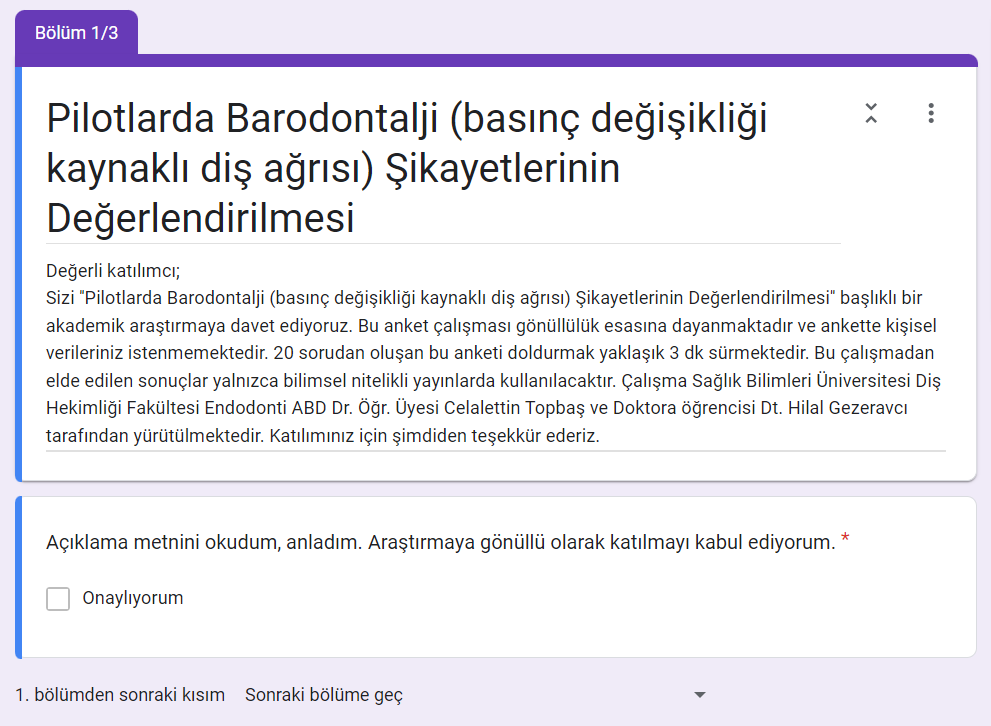

Supplement: Supplemental Information 2 [file peerj-12-17290-s002.png]
