# Supplementary material for: Relationships among barodontalgia prevalence, altitude, stress, dental care frequency, and barodontalgia awareness: a survey of Turkish pilots
Source: PeerJ. 2024 Apr 19;12:e17290. doi: 10.7717/peerj.17290 (PMC11034504; doi:10.7717/peerj.17290)
Supplement: Supplemental Information 4 [file peerj-12-17290-s004.docx]

Evaluation of Barodontalgia (toothache caused by pressure change) Complaints in Pilots

Dear participant

We would like to invite you to participate in an academic research titled "Evaluation of Complaints of Barodontalgia (toothache caused by pressure change) in Pilots". This survey is voluntary and your personal data is not requested. It takes approximately 3 minutes to complete this 20-question questionnaire. The results obtained from this study will only be used in scientific publications. The study was conducted under the supervision of University of Health Sciences, Faculty of Dentistry, Department of Endodontics, Dr. Lecturer. Prof. Dr. Celalettin Topbaş and PhD student Dt. Hilal Gezeravcı, PhD student. Thank you in advance for your participation.

* Required

I have read and understood the explanation text, and I agree to participate in the research voluntarily.

*Check all that apply.*

I approve

1. Gender *

*Check only one option.*

Female

Male

2. Age Range? *

*Check only one option.*

20 - 30

31 - 40

41 - 50

51 and above

3. What is your field of aviation? *

*Check only one option.*

Civilian

Military

4. Have you ever heard of barodontalgia? *

*Check only one option.*

Yes No

5. How often do you go to the dentist? *

*Check only one option.*

Every six months Once a year

Only in case of complaints

6. What is the treatment(s) performed during your visit? (You can select more than one option.)

*Check all that apply.*

No treatment

Filling

Canal treatment Tooth extraction Implant surgery

Tartar cleaning and Bleaching Prosthetic

Orthodontic Other

7. Have you ever stopped flying due to dental treatment? *

*Check only one option.*

Yes No

If you answered "NO" to Question 7;

8. Did you have any problems with your flights immediately after treatment?

*Check only one option.*

Yes No

9. Do you have the habit of clenching and/or grinding your teeth in your daily life? *

*Check only one option.*

Yes No

I don’t know

10. Do you clench your teeth during the flight? *

*Check only one option.*

Yes No

I don’t know

If you answered "YES" to Question-9 and/or Question 10;

11. Does your habit of clenching your teeth cause pain in your jaw? joint?

*Check only one option.*

Yes No

12. Have you ever experienced a toothache during a flight?

*Check only one option.*

Never Once

Twice

Three or more times

*If you answered "Never" to question 12, you can end the survey.

13. Please rate the intensity of the pain you experience during the flight on a scale of 1 to 5.

*Check only one option.*

0 - None

1 - Very mild 2 - Mild

3- Moderate

4- Severe

5- Very severe

14. How would you describe the pain you feel?

*Check only one option.*

Throbbing Tingling

15. How long did you feel the pain during the flight?

*Check only one option.*

None

A few seconds Two to three minutes

Ten to fifteen minutes

30 minutes and more

16. At which stage of the flight did you feel the pain? (You can select more than one option.)

*Check all that apply.*

None

Take-off Landing

Straight flight Continuous throughout the flight

17. In what altitude range did you complain of pain? (You can choose more than one option.)

*Check all that apply.*

None

0 - 2000 feet

2001 - 5000 feet

5001 feet and above

18. Did your complaint of pain go away immediately after the flight?

*Check only one option.*

None

Yes No

19. Have you ever been examined by a dentist after flight-related complaints?

*Check only one option.*

Never

Yes No

If you answered "YES" to Question-19;

20. According to the examination, what was the cause of your pain? (You can select more than one option.)

*Check all that apply.*

None

Not found. Caries

Old treatment (filling, Canal treatment…)

Bruxism

Abscess

Sinusitis

Orthodontic

Other:

[Form](https://www.google.com/forms/about/?utm_source=product&utm_medium=forms_logo&utm_campaign=forms)s
