# Supplementary material for: Relationships among barodontalgia prevalence, altitude, stress, dental care frequency, and barodontalgia awareness: a survey of Turkish pilots
Source: PeerJ. 2024 Apr 19;12:e17290. doi: 10.7717/peerj.17290 (PMC11034504; doi:10.7717/peerj.17290)
Supplement: Supplemental Information 5 [file peerj-12-17290-s005.docx]

Pilotlarda Barodontalji (basınç değişikliği kaynaklı diş ağrısı) Şikayetlerinin

Değerlendirilmesi

Değerli katılımcı;

Sizi ''Pilotlarda Barodontalji (basınç değişikliği kaynaklı diş ağrısı) Şikayetlerinin Değerlendirilmesi'' başlıklı bir akademik araştırmaya davet ediyoruz. Bu anket çalışması gönüllülük esasına dayanmaktadır ve ankette kişisel verileriniz istenmemektedir. 20 sorudan oluşan bu anketi doldurmak yaklaşık 3 dk sürmektedir. Bu çalışmadan elde edilen sonuçlar yalnızca bilimsel nitelikli yayınlarda kullanılacaktır. Çalışma Sağlık

Bilimleri Üniversitesi Diş Hekimliği Fakültesi Endodonti ABD Dr. Öğr. Üyesi Celalettin Topbaş ve Doktora öğrencisi Dt. Hilal Gezeravcı tarafından yürütülmektedir. Katılımınız için şimdiden teşekkür ederiz.

* Gerekli

Açıklama metnini okudum, anladım. Araştırmaya gönüllü olarak katılmayı kabul *

ediyorum.

*Uygun olanların tümünü işaretleyin.*

Onaylıyorum

1. Cinsiyet *

*Yalnızca bir şıkkı işaretleyin.*

Kadın Erkek

2. Yaş aralığınız *

*Yalnızca bir şıkkı işaretleyin.*

20 - 30

31 - 40

41 - 50

51 ve sonrası

3. Faaliyet gösterdiğiniz havacılık alanı nedir? *

*Yalnızca bir şıkkı işaretleyin.*

Sivil Askeri

4. Barodontalji kavramını hiç duydunuz mu? *

*Yalnızca bir şıkkı işaretleyin.*

Evet Hayır

5. Ne sıklıkta diş hekimi muayenesi oluyorsunuz? *

*Yalnızca bir şıkkı işaretleyin.*

Altı ayda bir Yılda bir

Sadece şikayetim olduğunda

6. Ziyaretinizde uygulanan tedavi/tedaviler nedir? (Birden fazla seçenek *

işaretleyebilirsiniz)

*Uygun olanların tümünü işaretleyin.*

Tedavi yapılmayan

Dolgu

Kanal tedavisi Diş çekimi İmplant cerrahisi

Diş taşı temizliği ve beyazlatma Protetik tedavi

Ortodontik tedavi Diğer

7. Dental tedavi nedenli hiç uçuşa ara verdiniz mi? *

*Yalnızca bir şıkkı işaretleyin.*

Evet Hayır

Soru-7'ye ''HAYIR'' dediyseniz;

8. Tedaviden hemen sonra yaptığınız uçuşlarda herhangi bir problem yaşadınız mı?

*Yalnızca bir şıkkı işaretleyin.*

Evet Hayır

9. Günlük yaşamınızda diş sıkma ve/veya gıcırdatma alışkanlığınız var mı? *

*Yalnızca bir şıkkı işaretleyin.*

Evet Hayır

Bilmiyorum

10. Uçuş esnasında dişlerinizi sıkıyor musunuz? *

*Yalnızca bir şıkkı işaretleyin.*

Evet Hayır

Bilmiyorum

Soru-9 ve/veya Soru-10'a ''EVET'' dediyseniz;

11. Diş sıkma alışkanlığınız çene ekleminizde ağrıya sebep oluyor mu?

*Yalnızca bir şıkkı işaretleyin.*

Evet Hayır

12. Uçuş esnasında hiç diş ağrısı yaşadınız mı?

*Yalnızca bir şıkkı işaretleyin.*

Hiç yaşamadım. Bir kez

İki kez

Üç kez ve daha fazla

*12. soruya "Hiç yaşamadım." dediyseniz, anketi sonlandırabilirsiniz.

13. Uçuş esnasında yaşadığınız ağrı şiddetini 1 - 5 arasında numaralandırınız.

*Yalnızca bir şıkkı işaretleyin.*

0 -Hiç

1 - Çok hafif 2 - Hafif

3- Orta

4- Şiddetli

5- Çok şiddetli

14. Hissettiğiniz ağrıyı nasıl tanımlarsınız?

*Yalnızca bir şıkkı işaretleyin.*

Zonklama şeklinde Sızlama şeklinde

15. Uçuş süresince ağrıyı ne kadar süreyle hissettiniz?

*Yalnızca bir şıkkı işaretleyin.*

Hiç

Birkaç saniye İki- üç dakika

On - onbeş dakika

Otuz dakika veya daha fazla

16. Uçuşun hangi aşamasında ağrıyı hissettiniz? (Birden fazla seçenek işaretleyebilirsiniz)

*Uygun olanların tümünü işaretleyin.*

Hiç

Kalkış İniş

Düz uçuş Sürekli

17. Hangi irtifa aralığında ağrı şikayetiniz oldu? (Birden fazla seçenek işaretleyebilirsiniz)

*Uygun olanların tümünü işaretleyin.*

Hiç

0 - 2000 fit

2001 - 5000 fit

5001 fit ve üstü

18. Uçuştan hemen sonra ağrı şikayetiniz geçti mi?

*Yalnızca bir şıkkı işaretleyin.*

Hiç

Evet Hayır

19. Uçuşa bağlı oluşan şikayetlerden sonra diş hekimi muayenesi oldunuz mu?

*Yalnızca bir şıkkı işaretleyin.*

Hiç

Evet Hayır

Soru-19'a ''EVET'' dediyseniz;

20. Muayene sonucunda belirlenen ağrı sebebiniz nedir? (Birden fazla seçenek işaretleyebilirsiniz)

*Uygun olanların tümünü işaretleyin.*

Hiç

Ağrı sebebi bulunamamıştır. Çürük

Yapılmış eski tedaviler (dolgu, kanal tedavisi vb.)

Bruksizm

Abse

Sinüzit

Ortodontik

Diğer:

[Formlar](https://www.google.com/forms/about/?utm_source=product&utm_medium=forms_logo&utm_campaign=forms)
